# Ontogenetic shifts in leaf biomass allocation in crop plants

**DOI:** 10.1093/nsr/nwae349

**Published:** 2024-09-30

**Authors:** Renfei Chen, Suping Xiao, Chuancong Dong, Shubin Xie, Liang Zhang, Fan Wu, Chengyi Tu, Quan-Xing Liu, Shaopeng Wang, Ülo Niinemets, Alan Hastings, Karl J Niklas, Jianming Deng

**Affiliations:** School of Life Science, Shanxi Normal University, China; School of Mathematics and Computer Science, Shanxi Normal University, China; State Key Laboratory of Herbage Improvement and Grassland Agro-Ecosystems, College of Ecology, Lanzhou University, China; State Key Laboratory of Herbage Improvement and Grassland Agro-Ecosystems, College of Ecology, Lanzhou University, China; State Key Laboratory of Herbage Improvement and Grassland Agro-Ecosystems, College of Ecology, Lanzhou University, China; State Key Laboratory of Herbage Improvement and Grassland Agro-Ecosystems, College of Ecology, Lanzhou University, China; School of Economics and Management, Zhejiang Sci-Tech University, China; Department of Environmental Science, Policy, and Management, University of California, Berkeley, USA; School of Mathematical Sciences, Shanghai Jiao Tong University, China; Institute of Ecology, College of Urban and Environmental Science, and Key Laboratory for Earth Surface Processes of the Ministry of Education, Peking University, China; Crop Science and Plant Biology, Estonian University of Life Sciences, Estonia; Department of Environmental Science and Policy, University of California, Davis, USA; Santa Fe Institute, USA; School of Integrative Plant Science, Cornell University, USA; State Key Laboratory of Herbage Improvement and Grassland Agro-Ecosystems, College of Ecology, Lanzhou University, China

Leaves are the primary photosynthetic organ of vascular land plants as they provide photosynthates that maintain plant growth and reproduction. The extent to which plants allocate biomass to their leaf construction strongly influences photosynthetic performance, net primary productivity [1] and thus ecosystem functions [2]. Consequently, biomass allocation patterns have been a central focus in ecology. As a simple metric of plant biomass allocation patterns, the leaf mass ratio (i.e. the quotient of leaf biomass and total plant biomass) typically (but not invariably) decreases over time during ontogeny [3,4]. However, contrasting patterns have been reported [5] based on direct measurements at different ontogenetic stages or using different proxies of ontogeny, such as total plant biomass and plant height. Classic optimal allocation theory cannot provide reasonable explanations for these contrasting empirical observations, whereas transient biomass allocation pattern theory can [6,7]. This new theoretical framework pays attention to biomass allocation patterns over relatively short time scales rather than asymptotic behavior over long time scales [6,7]. The importance of investigating transient plant biomass allocation patterns emerges from the fact that observations over different time scales reveal statistically significant differences among various plant systems, thereby leading to conflicting theories and unnecessary debate. For example, the time scales of forest trees differ by orders of magnitude from those of annual or biennial crops [6]. Consequently, it is reasonable to suggest that the effects of the transient dynamics of plant biomass allocation should be investigated in the context of the ontogenetic time frames characteristic of individual species and/or growth forms. In addition, different researchers have emphasized biomass allocation patterns among different plant organs, whereas theoretical treatments of transient changes have typically focused on populations or communities, which result in the difficulties of applying transient dynamic theory to biomass allocation patterns [7]. Thus, a theoretical gap still exists in understanding the mechanism(s) underlying how and why, over transient timescales, leaf mass ratios quantitatively vary in response to biotic factors such as plant competition during plant ontogeny. The issue is especially important for domesticated crops, which are species that can finish their life cycles in one year. Here, we develop a theoretical model based on scaling theory in tandem with deterministic population models to predict the effects of plant–plant interactions on the numerical values of leaf mass ratios (Appendix A.1 in the supplementary data). Empirical data drawn from diverse crop species are used to test this model. The goal of the study was to determine (i) whether and how the leaf mass ratio (leaf biomass divided by total plant biomass) varies during ontogeny in transient timescales (perturbations in days), and (ii) whether and how intraspecific plant interactions affect the transient dynamics of leaf biomass allocation patterns.

The results show that the leaf mass ratio differs between corn and soybean, and even for the same crop species planted in different years. For example, both empirical observations and simulations show that the leaf mass ratio of corn initially decreases and then increases regardless of whether the trajectories converged (i.e. the equilibrium state in the mathematical model corresponding to the optimal state in which plants maximize their growth rate at a particular ontogenetic stage) or not at the end of ontogeny (Fig. 1A–D). In the case of soybeans sowed in 2015, changes in the leaf mass ratio are more complex, with an initial decrease, a subsequent increase, followed by an increasing trend (Fig. 1E and F). In contrast, in the case of soybeans sowed in 2016, the leaf mass ratio initially increases and then decreases (Fig. 1G and H).

**Figure 1.**
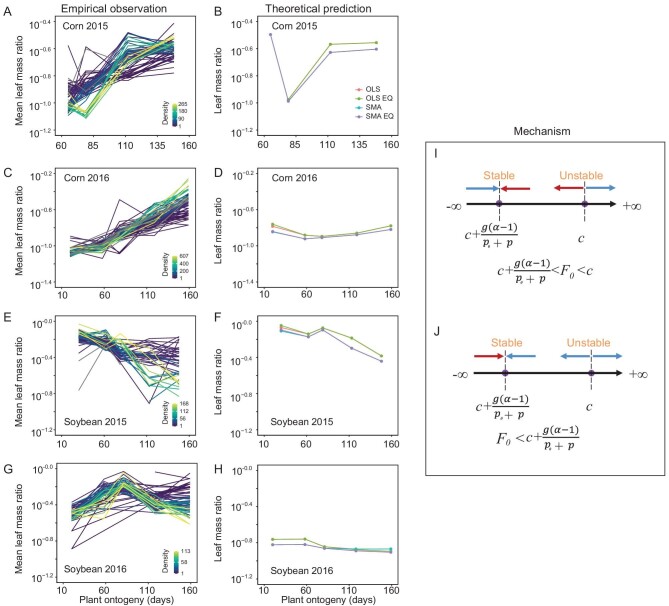
The dynamics of the leaf mass ratio in a one-species system predicted by theory and empirically observed under field conditions. Empirical variation of the leaf mass ratio as a consequence of ontogeny for corn planted in (A) 2015 and (C) 2016, and for soybean planted in (E) 2015 and (G) 2016. Plant ontogeny corresponds to the simulation time in the theoretical model. Density here denotes average value over time in the same plot. (B, D, F, H) The corresponding theoretical predictions of leaf mass ratio with parameter values of empirical estimated scaling exponent between leaf biomass and total biomass using statistical methods of both ordinary least squares (OLS) and standard major axis (SMA). The equilibrium states of leaf mass ratio are marked as ‘OLS EQ’ with the OLS statistical method, and ‘SMA EQ’ with the SMA statistical method. (I, J) Schematic diagram revealing the underlying mechanism that caused the perturbations of leaf mass ratio. During plant ontogeny, leaf mass ratio converges to the stable equilibrium state from different directions (the middle arrow in I and left arrow in J shown in the schematic diagram) depending on whether initial leaf mass ratio is larger (smaller) than the two equilibrium solutions of leaf mass ratio. In an ecological context, the mathematical equilibrium states correspond to plant development achieving maximum growth rate for each ontogenetic stage, during which allocation patterns do not experience additional increases in growth rates.

With different statistical methods (i.e. either ordinary least squares (OLS) or standard major axis (SMA); see Materials and Methods in the supplementary data), theoretical predictions of the variations in the leaf mass ratio based on empirically determined scaling exponents are robustly consistent (Fig. 1B, D, F, H). In addition, the leaf mass ratio in the equilibrium state consistently varies during ontogeny, and even overlaps in comparison with the dynamical leaf mass ratio (Fig. 1B, D, F, H). The underlying factor is the important role played by the initial conditions of the leaf mass ratio because it determines whether the trajectory will increase or decrease (Fig. 1I and J) (for details, see Appendix A.2 in the supplementary data).

These results show that the extent to which the leaf mass ratio varies (i.e. the magnitude of perturbations), on average, increases in response to strong intraspecific competition (Fig. S1A and C). However, the increasing trend is greatly weakened after detrending the time series (using the Augmented Dickey-Fuller test, the statistical *P* values are 0.52 and 0.01 before and after detrending, respectively), suggesting that both plant ontogeny and competition in tandem affect the variation of the leaf mass ratio (Fig. S1B and D).

Our work provides insights into the underlying mechanisms responsible for often starkly contrasting empirical observations. One meta-analysis shows that the leaf mass ratio of non-woody monocot and eudicot species decreases in response to plant ontogeny, whereas it initially increases and then decreases for woody gymnosperm and angiosperm species [3]. A superficial but direct explanation for this phenomenology is that the developmental differences between woody and non-woody species (particularly the production of secondary tissues in the former) result in contrasting differences in leaf mass ratio. However, these differences are observed even among woody species that do not appear to differ developmentally [8] and even within the same species [9]. These and other empirical observations indicate that new explanations are required to understand the mechanisms responsible for ontogenetic changes in the leaf mass ratio. From an integrated quantitative perspective, our model explains the contrasting results in natural systems by showing that the leaf mass ratio does not change in a simple monotonic way over the course of ontogeny but rather in a more complex manner that is sensitive to the initial conditions of the leaf mass ratio and subsequent plant–plant interactions as well as plant ontogeny, which are demonstrably far more complex and typically non-monotonic (Fig. 1, Fig. S1). By ‘initial conditions’ we mean the conditions occurring during the time of seedling emergence. For domesticated crop species, the initial conditions can change very quickly and make a significant difference even when occurring within a few days after germination. Therefore, the exact day at which measurements are taken may contribute both to the data used to study subsequent transient patterns of biomass allocation, and therefore to the extent to which different results are obtained by different researchers. In contrast, once the equilibrium state has been reached, plants achieve their maximum growth rate for each particular ontogenetic stage, after which plants achieve a steady state with no subsequent additional increase in their growth rate. However, plants cannot always achieve their equilibrium state and must therefore continue to alter their non-optimal biomass allocation pattern until the equilibrium state is achieved. In the parlance of our mathematical model, this is modeled (and empirically observed) as transient ‘perturbations’ around the equilibrium state. This phenomenon is most evident when the ability to change the biomass allocation pattern is much slower than the ontogenetic growth rate. Indeed, this phenomenology helps to explain why the predictions of the optimal allocation theory (which assumes that the equilibrium state has been, or can be achieved) are not always successful, and demonstrates the important role played by ontogenetic transient variations in plant biomass allocation.

Our model also emphasizes the importance of intra- and interspecific plant competition as well as ontogenetic changes in understanding changes in the leaf mass ratio. When the environment changes significantly, our model predicts that transient perturbations can be more prominent. Specifically, based on our analyses of the one species model, transient perturbations of the leaf mass ratio are intensified when the environment changes. For example, under relatively constant environmental conditions, the leaf mass ratio initially increases and then decreases when the initial leaf mass ratio is smaller than the value attained in the equilibrium state (Fig. 1J). Subsequently, the leaf mass ratio can drop to a very low level (smaller than both the equilibrium solutions) because of a sudden environmental change (such as drought, or severe cold). As a result, an iteration occurs, and the otherwise decreasing leaf mass ratio begins to increase and then decreases during the subsequent transient period. Future elaboration of our model requires a more careful and broader consideration of the effects of abiotic factors and the interplay between biotic and abiotic factors.

Our results are based on several important assumptions that must be clearly considered. We assume that conspecifics have the same life history strategy, and that the abiotic environment does not undergo a significant change over the course of plant ontogeny. These assumptions should be scrutinized by examining plant–plant interactions more broadly because trait ‘distinctiveness’ is clearly an emerging aspect of predicting the outcomes of plant competition [10]. In addition, we have thus far focused on simple one-species systems to test the theoretical predictions using empirical data from artificially controlled agricultural systems. In contrast, in natural communities such as natural forests or grasslands, many species coexist and experience sometimes significant changes in abiotic conditions. Future work must therefore extend our model to cope with multi-species communities experiencing realistic natural variations.

## Supplementary Material

nwae349_Supplemental_File
