# Supplementary material for: Ontogenetic shifts in leaf biomass allocation in crop plants
Source: Natl Sci Rev. 2024 Sep 30;11(10):nwae349. doi: 10.1093/nsr/nwae349 (PMC11498050; doi:10.1093/nsr/nwae349)
Supplement: nwae349_Supplemental_File [file nwae349_supplemental_file.docx]

Supplementary Materials for

**Ontogenetic shifts in leaf biomass allocation in crop plants**

Renfei Chen^1*^, Suping Xiao^2^, Chuancong Dong^3^, Shubin Xie^3^, Liang Zhang^3^, Fan Wu^3^, Chengyi Tu^4,5^, Quan-Xing Liu^6^, Shaopeng Wang^7^, Ülo Niinemets^8^, Alan Hastings^9,10^, Karl J. Niklas^11^ and Jianming Deng^3*^

*Corresponding author(s). E-mail(s): [dengjm@lzu.edu.cn](mailto:dengjm@lzu.edu.cn) ; [chenrf@sxnu.edu.cn](mailto:chenrf@sxnu.edu.cn)

**The file includes:**

Materials and methods

Appendix A.1 Derivation of theoretical models

Appendix A.2 Analytical solutions and stability analyses

Fig. S1

Table S1

# Materials and methods

## Model and analyses

Our theoretical framework focuses on the scaling relationship between leaf biomass *M_L_* and total plant biomass *M_T_* using the logistic growth dynamics equation because foliage leaves are the primary photosynthetic organ and because their biomass is positively correlated with metabolic rates and thus plant growth and ecology (*1, 11, 12*). Numerous studies have shown that the scaling of total leaf mass per plants scales as total plant mass conforms to a power function *M_L_* = *βM_T_^α^*, where *β* is the normalization constant and *α* is the scaling exponent (*11, 12*). Because total plant biomass includes standing leaf mass, it follows that the quotient of leaf mass and total plant mass, the leaf mass ratio (*M_LT_*) has a scaling exponent of *α* – 1. When log-transformed, the relationship between the leaf mass ratio and total plant biomass takes the form *F* = *c* + (*α* − 1)*T*, where *F* denotes *ln*(*M_LT_*), *c* denotes *ln*(*β*), and *T* denotes *ln*(*M_T_*). Prior work has shown that plant scaling relationships typically manifest log-log relationships (*13*). In addition, log-log transformation reduces skewness in regression residuals and improves normality. In turn, we use the widely accepted log logistic approach to model plant growth during ontogeny (*14, 15*).

Using the forgoing notation, the dynamics of the leaf mass ratio at time *t* in a single species system (Appendix A.1 in Supplementary Materials) is given by the equation

$\frac{dF}{dt}=\left(F-c\right)\left[g-\frac{\left(p_{0}+p\right)\left(F-c\right)}{\alpha-1}\right]$, (1)

where *g* is plant intrinsic growth rate, *p* denotes intraspecific competition, which is positively correlated with plant density *Q* and $p_{0}$ is the logistic constraints on plant growth when there is no plant-plant competition (See specific definitions of all symbols in Table S1).

Analyses show that Equ. (1) has two equilibrium states, i.e., *F*^∗^ = *c* and $F^{*}=c+\frac{g\left(\alpha-1\right)}{p_{0}+p}$ during plant ontogeny. Analyses also show that the equilibrium state (i.e., $F^{*}=c+\frac{g\left(\alpha-1\right)}{p_{0}+p}$) is stable, whereas *F*^∗^ = *c* is unstable. In an ecological context, the mathematical equilibrium states correspond to plant development achieving maximum growth rate for each ontogenetic stage, during which allocation patterns do not experience additional increases in growth rates. According to empirical observations (*16-18*), the scaling exponent *α* generally decreases from seedlings to maturity during plant ontogeny. Therefore, the stable equilibrium state $F^{*}$is predicted to gradually decrease ontogenetically. However, whether the leaf mass ratio increases or decreases depends on the derivative of the leaf fraction with respect to time (i.e., *dF/dt*). Specifically, the leaf mass ratio is expected to increase when *dF/dt* becomes positive, and decrease when *dF/dt* becomes negative. And, if the initial value of leaf mass ratio stays in between the two solutions, the leaf mass ratio always decreases. If, however the initial value is smaller than both solutions (which is usually the case in natural systems), the leaf mass ratio is predicted to increase until it surpasses the initially decreasing equilibrium point, i.e., $F^{*}=c+\frac{g\left(\alpha-1\right)}{p_{0}+p}$ and then decreases (See the schematic diagram in Fig. 1 I, J).

## Simulations

To investigate the transient dynamics of leaf mass ratios, we calculated the analytical solutions for the one species system using the dsolve function in MATLAB 2019a. Parameter values used in the theoretical models are estimated from the empirical observations on the crop species. To assess the robustness of the transient perturbations, parameter values of the scaling exponent of leaf biomass vs. total biomass are estimated under statistical approaches of both ordinary least squares (OLS) and standard major axis (SMA). As suggested by empirical observations, the scaling exponent of leaf biomass vs. total biomass approaches 1 for smaller plants, while it decreases to either 2/3 or 3/4 for relatively larger plants (*16-18*). Therefore, in one species system, we believe the scaling exponent of leaf vs. total biomass varies during plant ontogeny and is empirically estimated at different ontogenetic stage of the crop species we studied. To investigate the extent to which leaf mass ratios vary (i.e., magnitude of perturbations of leaf mass ratios), we simulated and calculated the coefficient of variation (CV) of leaf mass ratios during ontogeny in response to plant competition. CV was calculated using the equation

$CV=\frac{sd\left(e^{F_{T}}\right)}{mean\left(e^{F_{T}}\right)}$ , (6)

where $e^{F_{T}}$ represents all of the numerical values of leaf mass ratio during plant ontogeny, and *sd*() and *mean*() denote standard deviation and average of the focal interests, respectively. **Empirical data sampling**

All field experiments were performed in 2015, and 2016 at the Yuzhong Experimental Station, at the State Key Laboratory of Herbage Improvement and Grassland AgroEcosystems, Lanzhou University, Lanzhou, China. Soybean (*Glycine max (L.) Merr.*) and corn were singly sowed in the 1.5 m x 1.5 m plots with different densities to produce both low and high plant-plant competition. In addition, repeat treatments with 4 replications were performed to assess variability among the trials, during which plant height, basal stem diameters, and aboveground biomass were measured through destructive sampling between four to six times depending on the maturation time of different species after sowing in the spring. To reduce the effect of these destructive samplings and measurements on plant growth (especially when root systems are tangled), plant roots were sampled only during the last sampling events after which tracking measurements no more exist. Once individuals were sampled, they were immediately taken to the laboratory and oven dried at 115 °C for 30 min and subsequently at 65 ^o^C for 48-72 h to measure dry mass. During each sampling, we counted (rather than estimated) the abundance of individuals in each plot and the average value of plant density is used to evaluate the variations of leaf mass ratio during ontogeny under different gradients of plant density. For the high-sowing densities (used to simulate intense plant-plant competition), we randomly harvested at least ten individuals to measure height, basal stem diameter and biomass. For the low-sowing densities with minimum plant-plant competition, additional replicates were set up to ensure that a statistically sufficient number of individuals could be harvested to directly measure plant biomass at each specific ontogenetic stage.

**Data analyses**

For single sowed species, the effects of plant-plant interactions were comparatively easy to estimate using plant density as a proxy measure of plant-plant interactions. Plant height and basal stem diameter were used to estimate total plant volume and thus biomass in the cases where root biomass was not sampled. The parameters required to predict the relationships among plant height, basal stem diameter, and biomass were empirically estimated from the cases where root biomass was measured directly for the same species. To determine the variation in leaf mass ratios during ontogeny, the mean values of leaf fractions were calculated for individuals sampled in each plot. The trends in variation for each plot were then fitted using the loess (Local Polynomial Regression analyses) method in R package ggplot2. To evaluate transient perturbations, we calculated the coefficient of variation CV (*6*) of leaf mass ratios of the individuals sampled from each plot. To estimate the effect of plant-plant interaction on the variations of leaf mass ratio without ontogenetic trend, we perform Augmented Dickey-Fuller test with the function adf.test() in the R package “tseries” and detrending analyses using the R function diff(), To determine the numerical value of the leaf biomass vs. total plant biomass scaling exponent, the data for leaf biomass and total plant biomass were ln-transformed and subsequently regressed using the linear method within the function geom_smooth() in R package “ggplot2”.

**Data and materials availability**: All empirical data and simulation codes are available in the supplementary materials or the public repository https://github.com/RenfeiChencode/plant-biomass-allocation.

## Appendix A.1 Derivation of theoretical models

Based on the metabolic theory of ecology (*1, 11, 12*), the relationship between plant leaf biomass *M_L_* and total biomass *M_T_* is linked by the scaling exponent *α* and the coefficient *β*:

*M_L_* = *βM_T_^α^*. (A1)

With the quotient between leaf biomass and total biomass, we could achieve leaf biomass fraction *M_LT_*:

$M_{LT}=\frac{M_{L}}{M_{T}}=\beta M_{T}^{\alpha-1}$, (A2)

which can be log-transformed as:

*ln*(*M_LT_*) = *ln*(*β*) + (*α* − 1) *ln*(*M_T_*). (A3)

To be simple, we denote *F* = *ln*(*M_LT_*), *c* = *ln*(*β*), *T* = *ln*(*M_T_*). Then, we rewrite equation A3

*F* = *c* + (*α* − 1)*T* , (A4)

and thus:

$T=\frac{F-c}{\alpha-1}$. (A5)

Since the log logistic approach is widely used in ecology (*14, 15*), we use logistic model to study the dynamics of plant log-transformed total biomass during ontogeny. Therefore, we achieve the dynamics of plant total biomass (log-transformed, i.e., *T*) at time *t* (not log-transformed)

$\frac{dT}{dt}=gT-p_{0}T^{2}$,  (A6)

where *g* means intrinsic growth rate, and $p_{0}$ is a constant denotes the constraints in plant growth, which has a negative relationship with asymptotic plant total biomass. Based on equation A4, $\frac{dF}{dT}=\alpha-1$. Thus, without plant competition, the dynamics of leaf biomass fraction is

$\frac{dF}{dt}=\frac{dF}{dT}\frac{dT}{dt}=\left(\alpha-1\right)\left(gT-p_{0}T^{2}\right)$. (A7)

Based on equation A5, we have

$\frac{dF}{dt}=\left(F-c\right)\left[g-\frac{p_{0}\left(F-c\right)}{\alpha-1}\right]$.  (A8)

Under intraspecific competition, the dynamics of plant total biomass is

$\frac{dT}{dt}=gT-\left(p_{0}+p\right)T^{2}$ , (A9)

where $p=\delta Q$. $\delta$is a constant and *Q* denotes plant density which is positively related to plant intraspecific competition intensity. Similarly, the dynamics of leaf biomass fraction under intraspecific competition is

$\frac{dF}{dt}=\left(F-c\right)\left[g-\frac{\left(p_{0}+p\right)\left(F-c\right)}{\alpha-1}\right]$. (A10)

## Appendix A.2 Analytical solutions and stability analyses

Based on equation A10, we could achieve two equilibrium solutions (*F*^∗^) of leaf biomass fractions for the dynamics of one species model under intraspecific competition:

*F*^∗^ = *c*, (A17)

and

$F^{*}=c+\frac{g\left(\alpha-1\right)}{p_{0}+p}$. (A18)

To investigate the stability of the two solutions, we judge whether the value of *dF/dt* (denoting the variation rate of leaf biomass fractions in response to plant ontogeny) is positive or negative. If the initial value *F*_0_ of leaf biomass fractions is larger than the solution *F*^∗^ = *c* or smaller than the solution $F^{*}=c+\frac{g\left(\alpha-1\right)}{p_{0}+p}$ , the value of *dF/dt* is positive and thus plant leaf biomass fractions increase during plant ontogeny. Otherwise, it decreases. Therefore, if plant leaf biomass fractions stay in the vicinity of the solution $F^{*}=c+\frac{g\left(\alpha-1\right)}{p_{0}+p}$ , it converges to the solution and the solution is stable. On the contrary, if plant leaf biomass fractions stay in the vicinity of the solution *F*^∗^ = *c*, it shows divergency and the solution is unstable.

**References**

11. G. B. West, J. H. Brown, B. J. Enquist, A general model for the origin of allometric scaling laws in biology. *Science* **276**, 122–126 (1997).

12. J. H. Brown, J. F. Gillooly, A. P. Allen, V. M. Savage, G. B. West, Toward a metabolic theory of ecology. *Ecology* **85**, 1771–1789 (2004).

13. K. J. Niklas, Plant allometry: the scaling of form and process. University of Chicago Press. (1994).

14. R. H. Jones, R. R. Sharitz, Survival and growth of woody plant seedlings in the understorey of floodplain forests in South Carolina. *Journal of Ecology* **86**, 574–587 (1998).

15. C. T. Paine, T. R. Marthews, D. R. Vogt, D. Purves, M. Rees, A. Hector, L. A. Turnbull, How to fit nonlinear plant growth models and calculate growth rates: an update for ecologists. *Methods in Ecology and Evolution* **3**, 245–256 (2012).

16. D. S. Glazier, A unifying explanation for diverse metabolic scaling in animals and plants. *Biological Reviews* **85**, 111–138 (2010).

17. S. Mori, K. Yamaji, A. Ishida, S. G. Prokushkin, O. V. Masyagina, A. Hagihara, A. T. M. R. Hoque, R. Suwa, A. Osawa, T. Nishizono, T. Ueda, M. Kinjo, T. Miyagi, T. Kajimoto, T. Koike, Y. Matsuura, T. Toma, O. A. Zyryanova, A. P. Abaimov, Y. Awaya, M. G. Araki, T. Kawasaki, Y. Chiba, M. Umari, Mixed-power scaling of whole-plant respiration from seedlings to giant trees. *Proceedings of the National Academy of Sciences of The United States of America* **107**, 1447–1451 (2010).

18. T. Norin, A. K. Gamperl, Metabolic scaling of individuals vs. populations: evidence for variation in scaling exponents at different hierarchical levels. *Functional Ecology* **32**, 379–388 (2018).


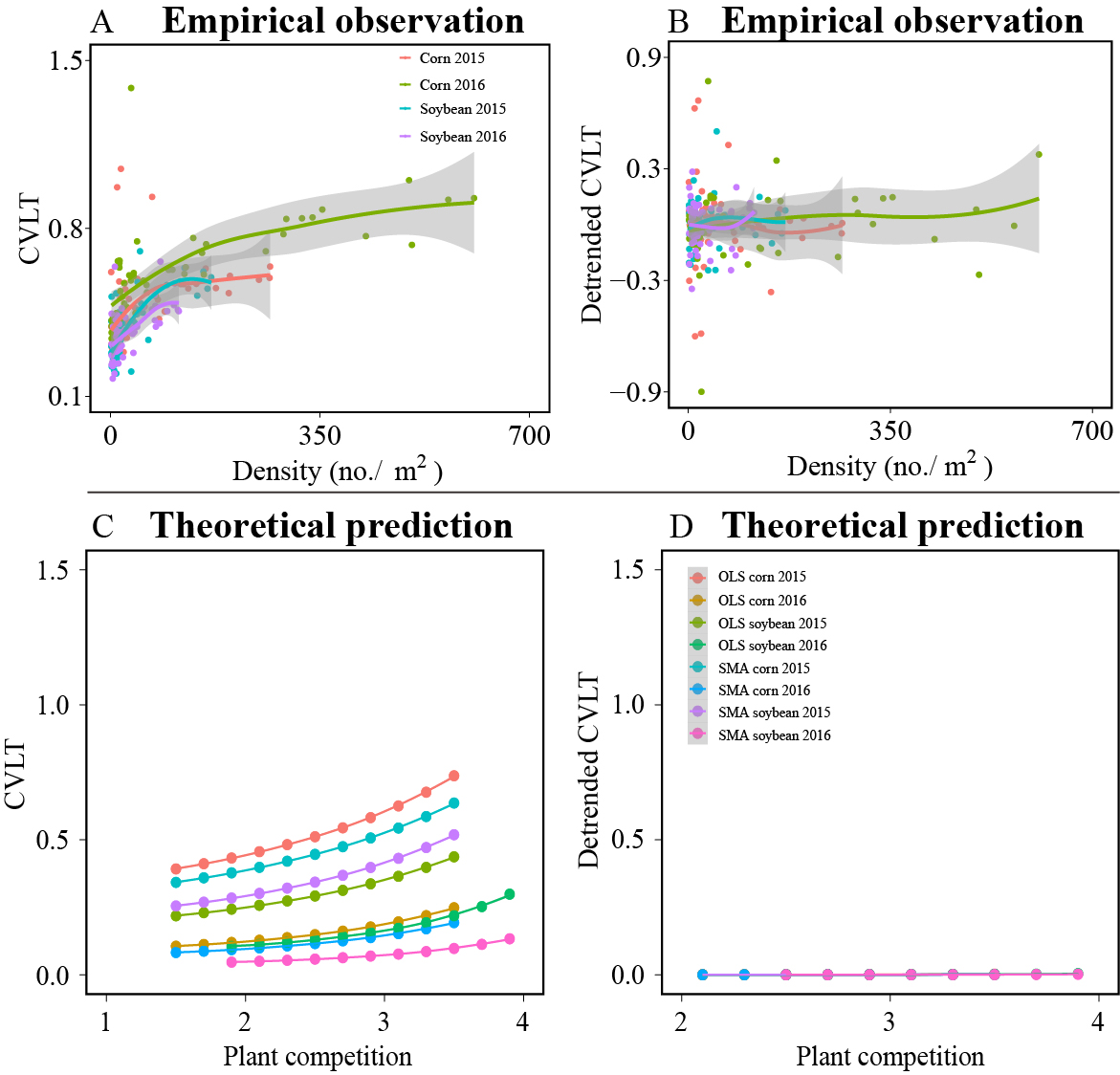


**Fig. S1** Variation of the leaf mass ratio in response to intraspecific competition observed under field conditions and predicted by theory. (A) Empirical variation of the coefficient of variation of leaf mass ratio (CVLT) in response to plant population density for corn and soybean planted in both 2015 and 2016. Density here denotes average value over time in the same plot. The shadow is 95% confidence interval in statistics. (B) The corresponding CVLT in response to plant density after removing the effect of time series trend through detrending analyses, which is marked as “Detrended CVLT”. (C, D) Theoretical effect of plant competition on both CVLT and Detrended CVLT with parameter values of empirical estimated scaling exponent between leaf biomass and total biomass using statistical methods of both ordinary least squares (OLS) and standard major axis (SMA) for both crop species.

**Table S1** Symbol definitions and parameter settings.

| Symbol | Description |
| --- | --- |
| *F* | Log-transformed leaf mass ratio |
| *F*∗ | Equilibrium state of log-transformed leaf mass ratio |
| *g* | Plant growth rate |
| *α* | Scaling exponent of leaf biomass vs. total biomass |
| *c* | Log-transformed scaling intercept |
| *p* | Plant competition |
| *t* | Time |
| CVLT | Coefficient of variation of leaf mass ratio |
